# Impact of newborn screening for SCID on the management of congenital athymia

**DOI:** 10.1016/j.jaci.2023.08.031

**Published:** 2024-01

**Authors:** Evey Howley, Zainab Golwala, Matthew Buckland, Federica Barzaghi, Sujal Ghosh, Scott Hackett, Rosie Hague, Fabian Hauck, Ursula Holzer, Adam Klocperk, Minna Koskenvuo, Nufar Marcus, Antonio Marzollo, Malgorzata Pac, Jan Sinclair, Carsten Speckmann, Maarja Soomann, Lynne Speirs, Sneha Suresh, Sophie Taque, Joris van Montfrans, Horst von Bernuth, Brynn K. Wainstein, Austen Worth, E. Graham Davies, Alexandra Y. Kreins

**Affiliations:** a Department of Immunology and Gene Therapy, Great Ormond Street Hospital for Children NHS Foundation Trust, London, United Kingdom; b San Raffaele Telethon Institute for Gene Therapy and Pediatric Immunohematology and Bone Marrow Transplantation Unit, IRCCS San Raffaele Scientific Institute, Milan, Italy; c Department of Pediatric Oncology, Hematology and Clinical Immunology, Medical Faculty, Center of Child and Adolescent Health, Heinrich-Heine-University, Düsseldorf, Germany; d University Hospitals Birmingham NHS Foundation Trust, Birmingham, United Kingdom; e Department of Paediatric Infectious Diseases and Immunology, Royal Hospital for Children, Glasgow, United Kingdom; f Department of Pediatrics, Dr von Hauner Children’s Hospital, University Hospital, Ludwig-Maximilians-Universität München, Munich, Germany; g University Children’s Hospital, Eberhard Karls University, Tübingen, Germany; h Department of Immunology, Second Faculty of Medicine, Charles University and University Hospital in Motol, Prague, Israel; i Division of Hematology-Oncology and Stem Cell Transplantation, New Children’s Hospital, University of Helsinki and Helsinki University Hospital, Helsinki, Finland; j Kipper Institute for Immunology, Schneider Children’s Medical Center of Israel, Petach Tikva, Israel; k Sackler Faculty of Medicine, Tel Aviv University, Tel Aviv, Israel; l Pediatric Hematology, Oncology and Stem Cell Transplant Division, Padua University Hospital, Padua, Italy; m Department of Immunology, Children’s Memorial Health Institute, Warsaw, Poland; n Starship Children’s Hospital, Auckland, New Zealand; o Institute for Immunodeficiency, Center for Chronic Immunodeficiency (CCI), Faculty of Medicine, Faculty of Medicine, Medical Center–University of Freiburg, Germany; p Center for Pediatrics and Adolescent Medicine, Department of Pediatric Hematology and Oncology, Faculty of Medicine, Medical Center–University of Freiburg, Germany; q Division of Immunology, University Children’s Hospital Zurich, University of Zurich, Zurich, Switzerland; r Department of Paediatrics, Royal Belfast Hospital for Sick Children, Belfast, United Kingdom; s Division of IHOPE, Department of Pediatrics, University of Alberta, Edmonton, Canada; t Department of Paediatrics, CHU Rennes, Rennes, France; u Department of Pediatric Immunology and Infectious Diseases, Wilhelmina Children's Hospital, University Medical Centre Utrecht, Utrecht, The Netherlands; v Department of Pediatric Respiratory Medicine, Immunology, and Critical Care Medicine, Charité Universitätsmedizin Berlin, Berlin, Germany; x Berlin Institute of Health, Charité Universitätsmedizin Berlin, Berlin, Germany; w Labor Berlin Charité-Vivantes, Department of Immunology, Berlin, Germany; y Berlin-Brandenburg Center for Regenerative Therapies, Berlin, Germany; z Department of Immunology and Infectious Diseases, Sydney Children’s Hospital, Sydney, Australia; aa School of Clinical Medicine, University of New South Wales, Sydney, Australia; bb Infection, Immunity and Inflammation Research and Teaching Department, University College London Great Ormond Street Institute of Child Health, London, United Kingdom

**Keywords:** Thymus transplantation, newborn screening, severe combined immunodeficiency, DiGeorge syndrome, athymia

## Abstract

**Background:**

Newborn screening (NBS) programs for severe combined immunodeficiency facilitate early diagnosis of severe combined immunodeficiency and promote early treatment with hematopoietic stem cell transplantation, resulting in improved clinical outcomes. Infants with congenital athymia are also identified through NBS because of severe T-cell lymphopenia. With the expanding introduction of NBS programs, referrals of athymic patients for treatment with thymus transplantation have recently increased at Great Ormond Street Hospital (GOSH) (London, United Kingdom).

**Objective:**

We studied the impact of NBS on timely diagnosis and treatment of athymic infants with thymus transplantation at GOSH.

**Methods:**

We compared age at referral and complications between athymic infants diagnosed after clinical presentation (n = 25) and infants identified through NBS (n = 19) who were referred for thymus transplantation at GOSH between October 2019 and February 2023. We assessed whether age at time of treatment influences thymic output at 6 and 12 months after transplantation.

**Results:**

The infants referred after identification through NBS were significantly younger and had fewer complications, in particular fewer infections. All deaths occurred in the group of those who did not undergo NBS, including 6 patients before and 2 after thymus transplantation because of preexisting infections. In the absence of significant comorbidities or diagnostic uncertainties, timely treatment was achieved more frequently after NBS. Treatment when younger than age 4 months was associated with higher thymic output at 6 and 12 months after transplantation.

**Conclusion:**

NBS contributes to earlier recognition of congenital athymia, promoting referral of athymic patients for thymus transplantation before they acquire infections or other complications and facilitating treatment at a younger age, thus playing an important role in improving their outcomes.

## Introduction

The introduction of newborn screening (NBS) for severe combined immunodeficiency (SCID) and T-cell lymphopenia is increasingly recognized as improving clinical outcomes for infants with SCID by promoting early initiation of protective and prophylactic measures, as well as early referral for corrective treatment with hematopoietic stem cell transplantation (HSCT).^1^, ^2^, ^3^ Delivery of HSCT before the age of 4 months results in high survival rates regardless of donor type.^3^^,^^4^ Infants with thymic aplasia and hypoplasia are also identified through these NBS programs, which are based on the enumeration of T-cell receptor excision circles on dried blood spots.^5^^,^^6^ Athymic infants require treatment with thymus transplantation, which is available at Great Ormond Street Hospital (GOSH) (London, United Kingdom)^7^ or Duke University Hospital (Durham, NC).^8^ Athymia is most commonly associated with complete DiGeorge syndrome due to 22q11.2 deletion syndrome (22q11.2DS) or coloboma, heart defects, choanal atresia, growth or mental retardation, genital hypoplasia, and ear anomalies and/or deafness (CHARGE) syndrome, but it has also been diagnosed in other rare disorders.^9^ Athymic patients frequently have syndromic comorbidities requiring acute medical attention and/or corrective surgery.^10^^,^^11^ In the absence of NBS, recognition of their SCID phenotype may be delayed, increasing their risk of infections and other complications before referral for thymus transplantation.^11^^,^^12^ Universal and pilot NBS programs are being implemented in an increasing number of countries.^13^ In October 2019, a 19-day-old patient with complete DiGeorge syndrome was the first infant identified by NBS to be referred for thymus transplantation at GOSH, receiving the procedure less than 4 weeks later.^6^ Since then, we have seen a steady increase in referrals for patients identified through NBS. Over the period from October 2019 to February 2023, a total of 44 patients were referred, including 19 infants (43%) diagnosed through NBS programs in 11 countries. The purpose of this brief report is to highlight the benefits of NBS for athymic patients in terms of timely diagnosis and referral for corrective treatment, including the improved kinetics of T-cell count recovery after thymus transplantation in younger patients.

## Results and discussion

**Fig 1:**
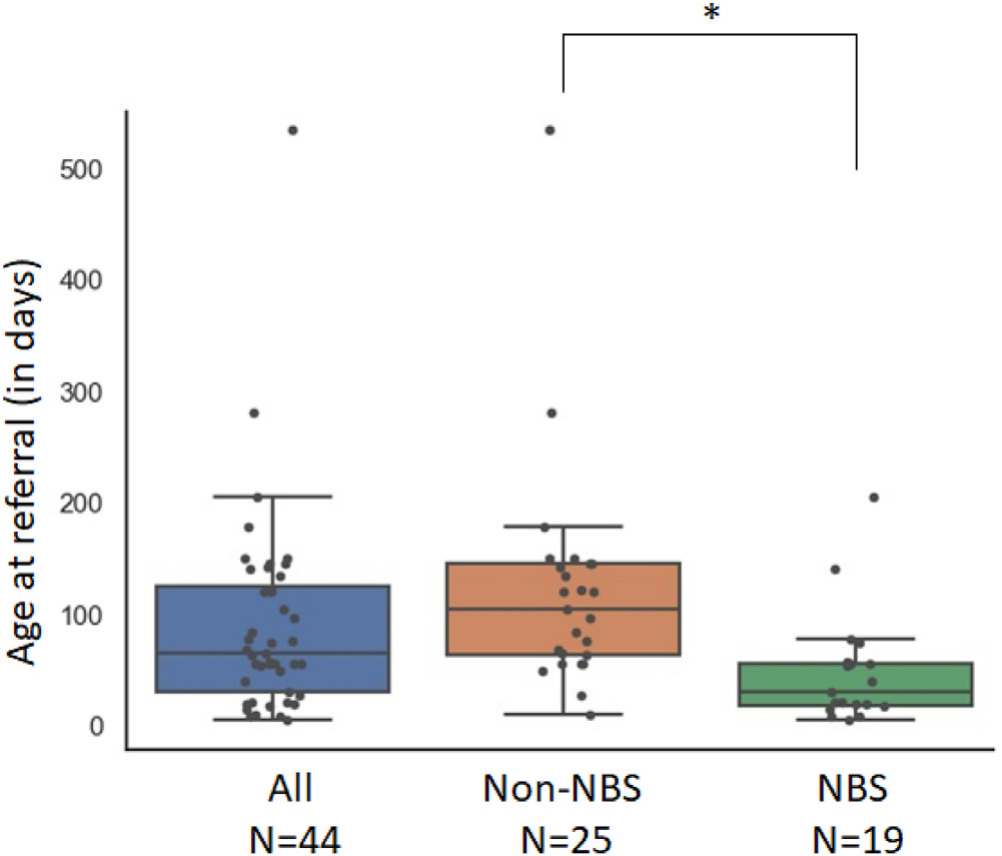
Age at referral for consideration of thymus transplantation. Box plots showing the differences in age at referral for 44 athymic infants identified after clinical presentation (n = 25) or diagnosed via an NBS program (n = 19). Age at referral is indicated in days. ∗*P* ≤ .001.

Among these 44 infants, 31 (71%) were diagnosed with congenital athymia due to 22q11.2DS (n = 17) or CHARGE syndrome (n = 14) and 7 were diagnosed with athymia due to rare thymic stromal cell defects, including TBX1 deficiency (n = 4), FOXN1 deficiency (n = 1), and PAX1 deficiency (n = 2) (Table I). No known defect previously associated with SCID or athymia was identified in the remaining 6 patients. The median age at referral for the 19 patients who had undergone NBS was 31 days (range 5-205 days) compared with 105 days (range 10-534 days) for the 25 patients diagnosed through clinical presentation (*P* ≤ .001 [according to the Mann-Whitney *U* test]) (Fig 1). At the time of referral, infants diagnosed through NBS had less invasive infections (n = 3 of 19 [16%]) than did the infants who did not undergo NBS (n = 12 of 25 [48%]) (*P* ≤ .05 [Fisher exact test]) (Table II). Two infants in the NBS group acquired postnatal cytomegalovirus infections, including 1 patient who presented clinically with hypocalcemic seizures at age 6 weeks after becoming lost to follow-up despite a positive NBS result. Six patients died of systemic viral infections before treatment (Table II); all of them were in the non-NBS group (n = 6 of 25 [24%]). Athymic patients are at risk of developing Omenn syndrome (OS) over time.^7^^,^^8^ Four of the 19 patients in the NBS group (21%) and 9 of the 25 patients in the non-NBS group (36%) had OS-like symptoms before thymus transplantation (Table II), including 1 patient in the non-NBS group who had progressive inflammatory disease and died before transplantation. Because of complex comorbidities, corrective treatment was not pursued in an additional 8 of 44 patients (18%), who were equally distributed between the NBS and non-NBS groups (Table II). At the time of data collection, 26 of 44 patients (59%) had received a thymus transplant (Table I), with an overall median referral-to-treatment time of 119 days (3.9 months) (range 25-392 days). Three recently referred patients are awaiting treatment. As a result of earlier referral, 40% of the infants in the NBS group (6 of 15) received a transplant before age 4 months compared with 18% of those in the non-NBS group (n = 2 of 11). Of the 9 of 15 patients (60%) in the NBS group who received a transplant at a later age (median 225 days [range 137-597 days]), 2 were treated at just (ie, at 17 and 22 days) over the cutoff of 4 months for logistical reasons, whereas the transplants of the other 7 were delayed for clinical reasons, including the need for surgery for congenital heart disease (n = 2) or respiratory tract anomaly (n = 1), and novel or undefined causes of T-cell lymphopenia (n = 4) requiring additional investigations and a period of observation to determine that the T-cell lymphopenia was due to athymia (Table I).

**Table I:** Athymic patients referred for thymus transplantation at GOSH

Diagnosis		No. of infants (N = 44)	No. identified by NBS (n = 19)	No. who received a transplant (n = 26)	No. who received a transplant at age <4 mo (n = 8)	Median age at transplantation (d), range	
cDGS		35 (80%)	14 of 35 (40%)	19 of 35 (54%)	7	142 (44-381)	
	22q11.2DS	17 (39%)	8 of 17 (47%)	12 of 17 (71%)	5	170 (44-301)	
	CHARGE syndrome	14 (32%)	6 of 14 (43%)	6 of 14 (43%)	2	133.5 (82-381)	
	TBX1 deficiency	4 (9%)	0 of 4 (0%)	1 of 4 (25%)	0	271	
Nude SCID	FOXN1 deficiency	1 (2%)	0 of 1 (0%)	0 of 1 (0%)	0	NA	
Other		8 (18%)	5 of 8 (62.5%)	7 of 8 (87.5%)	1	225 (110-597)	
	PAX1 deficiency	2 (4.5%)	1 of 2 (50%)	1 of 2 (50%)	0	338	
	Undefined∗	6 (13.5%)	4 of 6 (67%)	6 of 6 (100%)	1	217 (110-597)	

Summary of the number of patients identified by NBS and the number of athymic patients who received a transplant after being referred for thymus transplantation at GOSH with a diagnosis of complete DiGeorge syndrome, nude SCID, or other thymic stromal cell defects.

*cDGS*, Complete DiGeorge syndrome; *CHARGE*, Coloboma, heart defects, choanal atresia, growth or mental retardation, genital hypoplasia, and ear anomalies and/or deafness; *22q11.2DS,* 22q11.2 deletion syndrome; *NA*, not applicable.

∗ Including 4 patients with genetic defects under investigation and putatively associated with athymia.

**Table II:** Complications in athymic patients referred for consideration of thymus transplantation

Group	Infections	OS	Death before transplantation	Palliation for comorbidities
NBS (n = 19)	3∗ (15.7%)	4 (21%)	0	4 (21%)
Non-NBS (n = 25)	12† (48%)	9 (36%)	6 (24%)	4 (16%)

Overview of the number of patients with infections, OS, or life-limiting comorbidities at the time of referral for thymus transplantation, including the number of deaths before transfer for corrective treatment.

∗ Infections: cytomegalovirus (n = 2), parainfluenza virus type 3 (n = 1).

† Adenovirus (n = 3), parainfluenza virus type 3 (n = 1), rotavirus (n = 1), cytomegalovirus (n = 6), astrovirus (n = 1), and EBV (n = 1 in addition to cytomegalovirus).

**Fig 2:**
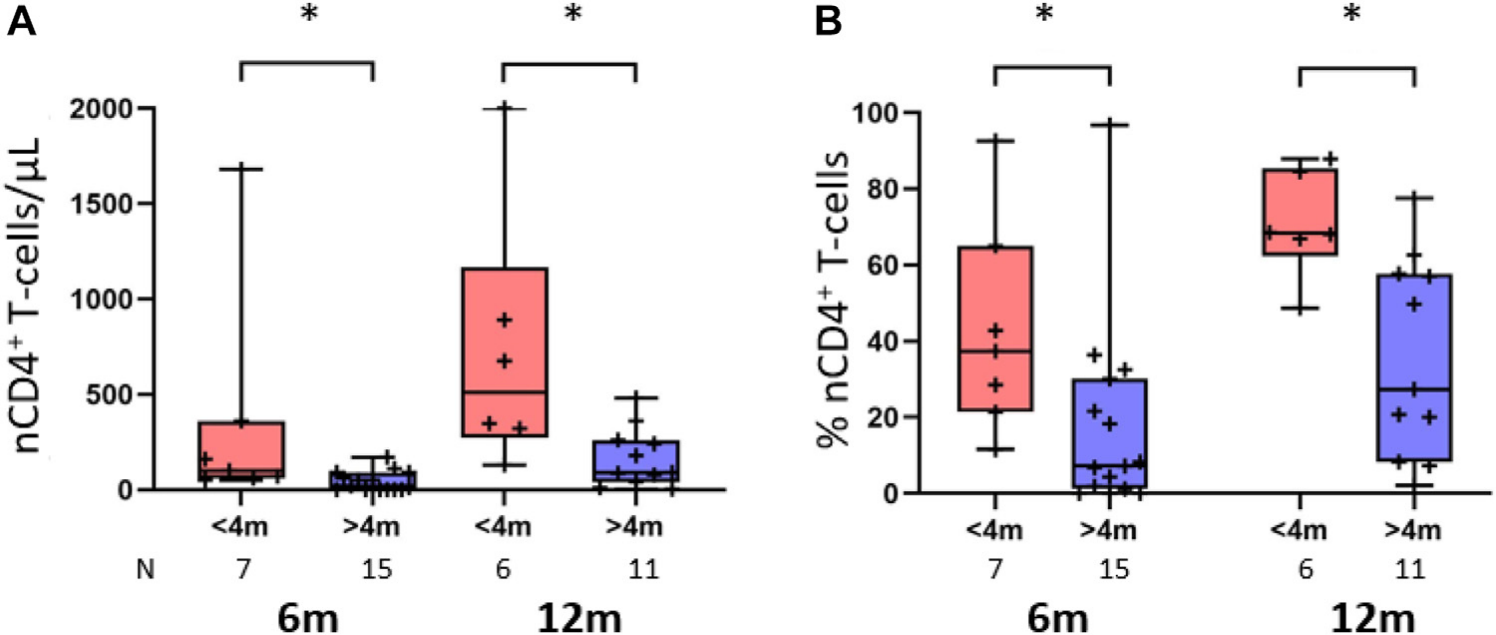
Thymic output at 6 and 12 months after thymus transplantation. Box plots showing the differences in absolute counts (**A**) and proportions of naive CD27^+^CD45RA^+^ T cells (percentage of CD4^+^ T cells) (**B**) at 6 and 12 months after treatment in patients who either underwent thymus transplantation when younger than 4 months or were treated when older than 4 months. ∗*P* ≤ .05. N indicates the number of patients with evaluable data at 6 ± 1 months and 12 ± 1 months.

With an overall median follow-up time after transplantation of 18 months (range 4-42 months) by May 2023, all but 3 patients are alive (n = 23 of 26 [88%]). All 3 deaths occurred among patients in the non-NBS group. Two patients had preexisting viral infections, dying at 8 and 9 months after thymus transplantation without establishment of thymopoiesis. The third patient had treatment-refractory autoimmune hemolytic anemia and died 3 years after transplantation from complications of immunosuppressive therapy. Regular immunologic monitoring was performed in all patients who received a transplant. Assessment of thymic output included measurement of CD4^+^CD27^+^CD45RA^+^ naive CD4^+^ T cells. At 6 plus or minus 1 months after transplantation (data available for 22 patients), the patients treated before age 4 months had higher absolute counts and proportions of naive CD4^+^ T cells (median 100 cells/μL [range 50-1680 cells/μL] and median 37.4% of CD4^+^ T cells [range 11.5%-92.6%]) than those treated when older than 4 months (median 20 cells/μL [range 0-170 cells/μL] and median 6.8% [range 0%-96.8%]), respectively (*P* = .011 and *P* = .017 according to the Mann-Whitney *U* test, respectively) (Fig 2). Because of a history of OS, 8 of 15 patients in the older group were receiving immunosuppression with cyclosporine compared with only 1 of 7 patients in the younger group. At 12 plus or minus 1 months after transplantation (data available for 19 patients), only 1 patient in the older group was still undergoing weaning cyclosporine treatment. Two patients with autoimmune complications, both in the older group, were receiving steroids and were excluded from this analysis. Thymic output increased in both groups (Fig 2). Absolute counts and proportions of naive CD4^+^ T cells remained higher in patients treated when younger than 4 months (median 334 cells/μL [range 130-2000 cells/μL] and median 68.5% [range 48.7%-87.9%]) than in those treated when older than 4 months (median 90 cells/μL [range 4-480 cells/μL] and median 27.4% [range 2.15%-77.5%]) (*P* = .015 and *P* = .010 [according to the Mann-Whitney *U* test], respectively) (Fig 2). Three patients in the older group did not show any evidence of beginning thymopoiesis at 12 months after transplantation (Fig 2, *B*). All 3 patients developed viral infections (cytomegalovirus in 2 and EBV in 1). The differences in thymic output between the 2 groups remained statistically significant after exclusion of these 3 patients (data not shown). Where measured, the counts of CD4^+^CD45RA^+^CD31^+^ recent thymic emigrants and levels of T-cell receptor excision circles per 10^6^ T cells, as additional parameters of thymic output, were consistent with the numbers of naive CD4^+^ T cells (data not shown). Of the 18 patients who underwent transplantation with at least 12 months of follow-up, 12 (67%) have discontinued immunoglobulin replacement treatment after recovery of satisfactory T-cell counts.

Overall, these results suggest that treatment at a younger age is associated with better immune reconstitution thanks to more efficient initial thymopoiesis and greater recovery of T-cell numbers after thymus transplantation.

Both centers offering thymus transplantation have reported an overall survival rate of 75% to 80%.^7^^,^^8^ Infections before immune reconstitution are the main cause of death. Despite NBS, the Primary Immune Deficiency Treatment Consortium reported infections before HSCT in 55% of patients with SCID.^14^ We also see infections in 16% of athymic children identified through NBS. Nevertheless, these patients benefit from close monitoring and early interventions, increasing their chances of a successful outcome despite preexisting infections. In Europe, coverage by NBS for SCID is still limited, and existing programs are in their infancy, with implementation differing across and within countries.^6^^,^^15^ Even with NBS, there is still a risk of infants being lost to follow-up and developing serious infection,^6^^,^^14^^,^^15^ as seen in our series. Identification of strategies to further reduce the incidence of infections in infants diagnosed through NBS will improve outcomes for athymic infants. Systemic viral infections, in particular, remain challenging because recovery of T-cell immunity after transplantation typically requires several months.^7^^,^^8^ Patients who develop OS before thymus transplantation require immunosuppressive treatment with cyclosporine and antithymocyte globulin.^7^^,^^8^ This has not been reported to adversely affect clinical outcomes after transplantation, but avoiding this complication with earlier corrective treatment has multiple benefits, including a reduced risk of drug toxicity, shorter hospital inpatient stays with a reduction in health care costs, and improved patient and family experience.

Congenital athymia is characterized by profound T-cell lymphopenia and absent thymic output.^7^, ^8^, ^9^ Absolute CD3^+^ T-cell counts are generally less than 50 cells/μL, unless patients have developed OS-like features, which are associated with higher CD3^+^ T-cell counts. The proportion of naive T cells, a measure of thymic output, is negligible (<5% of T cells) in all athymic patients. Circulating naive T cells appear 5 to 6 months after treatment and increase progressively over time, but they typically remain subnormal.^7^^,^^8^ In the context of the expanding implementation of NBS for SCID, athymic patients have increasingly been treated at a younger age at GOSH, and we aimed to investigate the impact of this on thymic output. Here, we have shown for the first time that treatment at a younger age (<4 months) is associated with higher thymic output in the first year after transplantation. Whether earlier treatment results in superior thymic output sustained over time and overall better immunologic outcomes will need to be confirmed through long-term follow-up of this growing cohort of patients.

Thymus transplantation should be undertaken as soon as possible; however, the recommendation for proceeding with corrective treatment by age 4 months, as in infants with SCID with hematopoeitic cell–intrinsic defects identified through NBS, should not be the benchmark for timely delivery of thymus transplantation in all infants. As seen in this brief report, athymic patients often have major comorbidities.^10^^,^^11^ Palliative care is considered and provided in patients with life-limiting comorbid conditions, including severe complex heart defects and severe neurologic impairment. For patients in whom thymus transplantation can be lifesaving, it may be necessary to first proceed with other procedures to achieve clinical stability.^11^ Furthermore, despite increasing access to next-generation sequencing, a significant number of patients with an SCID phenotype do not have a genetic variant in any of the known SCID genes.^16^ Infants with genetically undefined T^–^B^+^NK^+^ SCID and T-cell lymphopenia require significant additional diagnostic work, including broader genetics and *ex vivo* T-cell differentiation assays before a therapeutic decision can be made.^17^, ^18^, ^19^ Although most patients have hematopoietic cell–intrinsic defects, which can be treated by HSCT, an increasing number of patients are found to have congenital athymia and require thymus transplantation. In some infants, particularly those with novel thymic stromal cell defects with variable penetrance, whether athymia is complete may not be immediately clear, and a period of observation may be required to determine whether thymus transplantation is indicated.^11^ For these multiple reasons, delay in treatment may be necessary despite early diagnosis on NBS.

In conclusion, our experience over the past 3 years highlights the benefits of NBS for athymic patients, owing to earlier recognition and referral for thymus transplantation before acquisition of infections or development of other complications. Despite the fact that GOSH is the only thymus transplantation center in Europe, it provides accessible, timely treatment, and in our cohort, earlier treatment has been associated with more efficient early thymopoiesis and immune reconstitution.

This work was completed under research ethics approval from the London Bloomsbury Research Ethics committee (07/Q0508/43).

## Disclosure statement

Supported by LetterOne in conjunction with GOSH Children’s Charity (to E.H., Z.G., E.G.D., and the 10.13039/501100000765University College London (UCL) 10.13039/501100001282Great Ormond Street Hospital [GOSH] thymus transplantation program); the 10.13039/501100009553Czech Health Research Council and 10.13039/100009647Ministry of Health, Czech Republic (grants NU20-05-00282 and NU23-05-00097 [to A.K.]); and the 10.13039/100010269Wellcome Trust (grant 222096/Z/20/Z [to A.Y.K.]). All research at GOSH is supported by the UK National Institute of 10.13039/100005622Health Research and Great Ormond Street 10.13039/100014461Biomedical Research Centre.

Disclosure of potential conflict of interest: The authors declare that they have no relevant conflicts of interest.

**Clinical implications**

NBS facilitates early diagnosis of congenital athymia and timely treatment before patients develop complications. Treatment at a younger age is associated with quicker immune reconstitution thanks to earlier thymopoiesis and increase in T-cell counts after thymus transplantation.
